# Subclinical Carotid Atherosclerosis and Reduced DAD Score for Cardiovascular Risk Stratification in HIV-Positive Patients

**DOI:** 10.5935/abc.20190227

**Published:** 2020-01

**Authors:** Achilles Gustavo Silva, Rafael Vieira Paulo, Mario León Silva-Vergara

**Affiliations:** 1 Universidade Federal do Triangulo Mineiro, Uberaba, MG - Brazil

**Keywords:** Carotid Artery Diseases, HIV, Acquired Immunodeficiency Syndrome/complications, Indicators of Morbidity, Antiretroviral Therapy, Highly Active, Risk Factors

## Abstract

**Background:**

HIV-positive patients are twice as likely than the general population to have a heart attack and are four times at greater risk of sudden death. In addition to the increased risk, these individuals present with cardiovascular events on average approximately 10 years earlier than the general population.

**Objective:**

To compare Framingham and reduced DAD (Data Collection on Adverse Effects of Anti-HIV Drugs Cohort) scores for cardiovascular risk assessment in HIV-positive patients and potential impact on clinical decision after evaluation of subclinical carotid atherosclerosis.

**Methods:**

Seventy-one HIV-positive patients with no history of cardiovascular disease were clinically evaluated, stratified by the Framingham 2008 and reduced DAD scores and submitted to subclinical carotid atherosclerosis evaluation. Agreement between scores was assessed by Kappa index and p < 0.05 was considered statistically significant.

**Results:**

mean age was 47.2 and 53.5% among males. The rate of subclinical atherosclerosis was 39.4%. Agreement between scores was 49% with Kappa of 0.735 in high-risk patients. There was no significant difference between scores by ROC curve discrimination analysis. Among patients with intermediate risk and Framingham and reduced DAD scores, 62.5% and 30.8% had carotid atherosclerosis, respectively.

**Conclusion:**

The present study showed a correlation between the scores and medium-intimal thickening, besides a high correlation between patients classified as high risk by the Framingham 2008 and reduced DAD scores. The high prevalence of carotid atherosclerosis in intermediate risk patients suggests that most of them could be reclassified as high risk.

## Introduction

Currently, about 36.7 million people are infected with HIV worldwide, and 1.8 million cases are diagnosed every year, while 1 million deaths occur.^1^ In Brazil, estimates say that 813,000 people are infected with HIV, with 48,000 new cases and 14,000 deaths in 2016.^2^ Over the last decades, the use of antiretroviral therapy (ART) has led to a progressive decrease in mortality caused by opportunistic diseases and, consequently, there has been a considerable increase in the survival of these patients. AIDS has become a chronic disease and permanent immune activation, caused by the HIV virus, which translates into a systemic inflammatory process with significant repercussions in various organs and systems, especially cardiovascular system, brain, kidneys and bones, which leads to premature aging.

Cardiovascular diseases emerged as an important cause of morbidity and mortality in this group of patients. Data from the DAD study (Data Collection on Adverse Effects of Anti-HIV Drugs), published in 2014, indicate that 11% of deaths of HIV-positive patients are caused by cardiovascular diseases.^3,4^ HIV-infected patients are at twice as high risk of having a heart attack than the general population and four times more likely to have sudden death.^5,6^ In addition to the increased risk, people with HIV experience cardiovascular events approximately 10 years before the general population, on average.^7^

Although traditional cardiovascular risk scores, such as Framingham, have been developed for the general population, their use in HIV-positive patients is not well defined.^8^ Based on the prospective multicenter DAD study, which was a collaboration of 11 cohorts of HIV-positive patients treated at 212 clinics in the United States, Europe, Argentina and Australia, algorithms were developed specifically for this population. The DAD score was first published in 2010, and considered CD4 count, use of Abacavir, and time of exposure to protease inhibitors and nucleoside reverse transcriptase inhibitors in addition to classic cardiovascular risk factors.^9^ In order to simplify risk stratification of HIV positive patients and due to the difficulty of assessing previous antiretroviral therapy regimens, a modification in the DAD score was proposed and published in 2016, assessing the same clinical outcomes in 5 years, but not using the classes and time of exposure to ART.^10^

The aim of this study was to compare the Framingham and reduced DAD scores for cardiovascular risk assessment in HIV-positive patients and the potential impacts on clinical decision after evaluation of subclinical carotid atherosclerosis.

## Methods

### Population

Seventy-one asymptomatic HIV-positive patients on regular ART, with no previous diagnosis of cardiovascular disease, and in regular follow-up at the Infectious and Parasitic Diseases Outpatient Clinic of *Universidade Federal do Triângulo Mineiro* (UFTM), in Uberaba, Minas Gerais, were included.

### Clinical assessment

Clinical, demographic and anthropometric data were obtained by clinical interview and included risk factors for cardiovascular disease, namely: age (≥ 45 years in men and ≥ 55 years in women), smoking (current use or cessation in the last 30 days), family history of early coronary artery disease (CAD) (myocardial infarction or death from CAD in first-degree relatives, if men < 55 years and women < 65 years), systemic arterial hypertension (previous diagnosis with medication for hypertension and/or blood pressure >140x90 mmHg), dyslipidemia (previous diagnosis with use of lipid lowering drugs and/or laboratory abnormalities according to current guidelines and described in the following), diabetes mellitus (previous diagnosis with use of blood glucose lowering medications and/or blood glucose monitoring > 126 mmHg). Body mass index (BMI) was calculated as the ratio between weight in kilograms and height squared in meters and considered normal from 18.5 to 24.9 kg/m^2^, overweight from 25.0 to 29.9 kg/m^2^ and obesity as ≥30.0. Waist circumference was measured in centimeters at the level of the umbilical scar and considered abnormal according to the International Diabetes Federation (IDF)’s metabolic syndrome standards.^11^

Blood pressure was measured during clinical evaluation at the outpatient clinic using an OMRON automatic arm blood pressure measuring device (HEM-7113), in compliance with current guidelines for systemic arterial hypertension, and the each individual’s level of activity was assessed by the short version of IPAQ (International Questionnaire on Physical Activity), with those who reported performing physical activity lasting < 10 minutes per week being considered sedentary.

### Laboratory assessment

All patients had 12-hour fasting peripheral venous puncture blood collection. Blood counts, blood glucose (RV = 60 to 99 mg/dL), total cholesterol, triglycerides, HDL-cholesterol, LDL-cholesterol, urea (RV ≤ 50 mg/dL), creatinine (RV = 0.4 to 1,4 mg/dL), sodium (RV = 136 to 145 mmol/L), potassium (RV = 3.5 to 5.1 mmol/L). Blood glucose, total cholesterol, LDL-cholesterol and triglycerides were considered altered if > 100 mg/dL, 200 mg/dL, 160 mg/dL and 150 mg/dL, respectively, and HDL-cholesterol was considered low when < 40 mg/dL in men and < 50 mg/dL in women.

The blood samples were processed at the Laboratory of Clinical Analysis of the UFTM Clinics Hospital. Total cholesterol, HDL-cholesterol and triglycerides were determined by colorimetric-enzymatic method in a Roche Cobas 6000 apparatus. LDL-cholesterol was calculated by the formula [(total cholesterol-HDL-cholesterol) - (triglycerides/5)].

### Risk stratification

Estimates of cardiovascular risk were measured by the reduced DAD and Framingham 2008 scores. Framingham 2008 considers outcomes such as cardiovascular death, CAD, stroke, heart failure and claudication in 10 years, whereas simplified DAD includes acute myocardial infarction, stroke, coronary and carotid interventions and cardiovascular death in 5 years. According to the Framingham 2008 score, event rate was considered low risk when < 10%, intermediate risk when >10% and <20%, and high risk when >20%. For DAD, values <1% were considered low risk, 1% to 5% moderate risk, 5% to 10% high risk, and >10% very high risk.^9,12^ The simplified DAD score was calculated by means of a tool available at https://www.chip.dk/Tools-Standards/Clinical-risk-scores.

### Evaluation of subclinical atherosclerosis

Exams were performed at the Radiology Department of the UFTM Clinical Hospital with a Toshiba Aplio 400 ultrasound device using a 10-14 MHz linear and multifrequency probe. Patients were evaluated in supine position, in a semi-dark room, with their neck positioned at 45º. The distal portions of the right and left common carotid arteries (1 cm before bifurcation) and proximal segments (2 cm) of the internal carotids were evaluated. The medium-intimal complex (MIC) was measured by the distance between two echogenic lines, the lumen-intima interface and media-adventitia interface, on the vessel’s posterior wall. The MIC was considered thickened if > 0.8 mm in the common carotid artery and the presence of plaques was established by a focal structure extending at least 0.5 mm to the vessel lumen and/or measuring more than 50% of the adjacent MIC value, and/or MIC greater than 1.5 mm.^13^

### Statistical analysis

Qualitative variables were expressed by frequency distribution and quantitative variables with normal distribution, as per the Kolmogorov-Smirnov test, were expressed as mean and standard deviation, and those with non-Gaussian distribution as median and interquartile range. The correlations in which the variables had non-Gaussian distribution were evaluated by the Spearman’s coefficient. Agreement between scores was assessed by Kappa index and discrimination power of scores was assessed by C-statistics, defined by the area below the ROC curve relating to the finding of subclinical atherosclerosis. The statistical software GraphPad Prism version 5 was used in the process. Statistical significance was set at p < 0.05.

## Results

From January 2016 to July 2017, 71 HIV patients under regular treatment were evaluated. All patients had an undetectable viral load, and had been in ART for over a year, asymptomatic and with no history of cardiovascular disease. Mean age was 47.23 ± 9.36m with 53.52% of the sample being composed of male patients, median time to HIV infection diagnosis of 12 years (6-17), and CD4 lymphocyte count of 654, 6 ± 308.3 cells/mm^3^. The metabolic profile of these patients is shown in Table 1. We highlight the presence of alterations in triglycerides > 150 mg/dL or total cholesterol >200 mg/dL in 41 (57.74%) cases.

**Table 1:** Main epidemiological, clinical and laboratory aspects of 71 HIV-positive patients

	n = 71
Males, n (%)	38 (53.52)
Age, years	47.23 ± 9.36
Time of HIV diagnosis (years)	12 (6-17)
Value of CD4 cells/mm^3^	654.6 ± 308.3
Weight, Kg	73.14 ± 16.37
BMI, Kg/m^2^	26.77 ± 5.21
Systolic pressure, mmHg	119.9 ± 15.47
Diastolic pressure, mmHg	75.97 ± 10.46
Total cholesterol, mg/dL	199.9 (171.2-244.9)
LDL, mg/dL	126.4 ± 40.27
HDL, mg/dL	47.85 ± 14.36
Triglycerides, mg/dL	169 (96-232)
Glycemia, mg/dL	100 (90.9-112.1)
Glycemia > 100, n (%)	35/69(50.72)
Triglycerides > 150, n (%)	38(53.52)
Low HDL, n (%)	26(36.61)
Triglycerides > 150 or total cholesterol > 200, n (%)	41(57.74)

HDL: high-density lipoprotein; BMI: body mass index; LDL: low-density lipoprotein; n: number of subjects.

**Figure 1:**
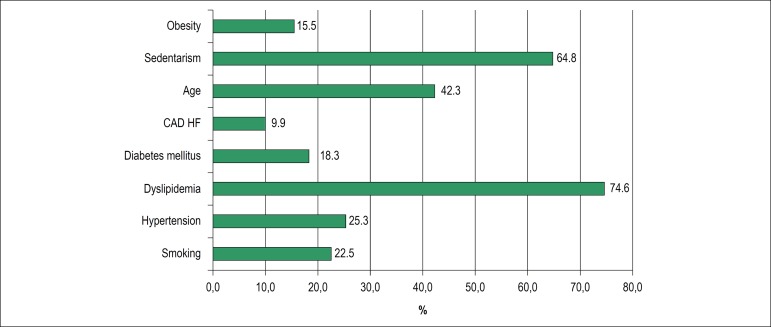
Cardiovascular risk factors in 71 HIV-positive patients.

Among the classic cardiovascular risk factors evaluated, the most frequent were dyslipidemia, physical inactivity and age in 53 (74.6%), 46 (64.78%) and 30 cases (42.25%), respectively (Figure 1). Increased waist circumference was found in 51 (71.83%) cases and metabolic syndrome, as defined by the IDF criteria, was found in 32 (45.07%) cases.

**Figure 2:**
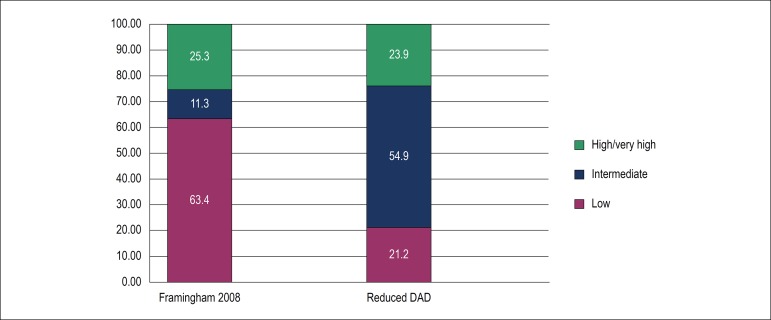
Cardiovascular risk rating in 71 HIV-positive patients according to Framingham 2008 and reduced DAD scores.

**Figure 3:**
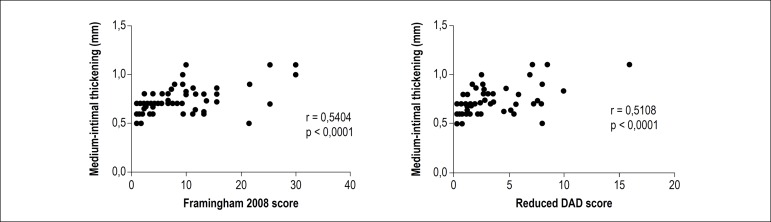
Correlation between medium-intimal thickening in 71 HIV-positive patients according to the Framingham 2008 and reduced DAD scores.

Cardiovascular risk stratification was made by the Framingham 2008 and reduced DAD scores, and results are shown in Figure 2. The identification of high and very high risk was similar in both scores, differing in other categories, as Framingham 2008 showed 63,4% of low risk cases and DAD score revealed 54.9% intermediate risk cases. When the degree of agreement between scores was evaluated, an overall Kappa index of 0.318 was observed with p < 0.001. However, there was stronger agreement for patients classified as high risk, lower agreement for low risk patients, and no statistically significant difference for intermediate risk subjects (Table 2). Both scores showed a statistically significant and positive correlation with the medium-intimal thickening (Figure 3).

**Table 2:** Degree of agreement between cardiovascular risk scores in 71 HIV-positive patients.

	Low	Intermediate	High
Category Kappa	0.268	0.084	0.735
p-value of category Kappa	0.001	0.226	< 0.001
95%CI of category Kappa	0.11 a 0.427	-0.052 a 0.22	0.502 a 0.967

95%CI: 95% confidence interval.

**Figure 4:**
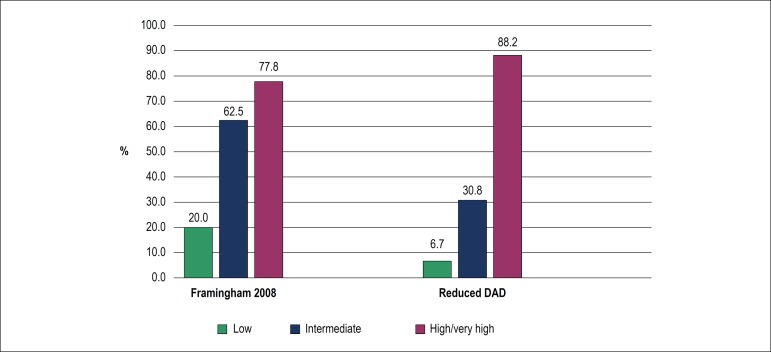
Frequency of subclinical atherosclerosis in 71 HIV-positive patients according to the Framingham 2008 and reduced DAD scores.

The medium-intimal thickening (highest thickness) in these patients was 0.73 ± 0.14 and there were 28 cases of subclinical atherosclerosis (39.4%). Of these, 17 (60.7%) patients presented non-significant plaque, 6 (21.4%) only thickening, and 5 (17.8%) had both plaque and thickening. In patients classified as high risk, the occurrence of subclinical atherosclerosis was 77.8% for the Framingham 2008 score and 88.2% for the reduced DAD score. In patients classified as low or intermediate risk, the rate of subclinical atherosclerosis was higher for Framingham 2008, with 20% of patients classified as low risk presenting subclinical atherosclerosis (Figure 4).

**Figure 5:**
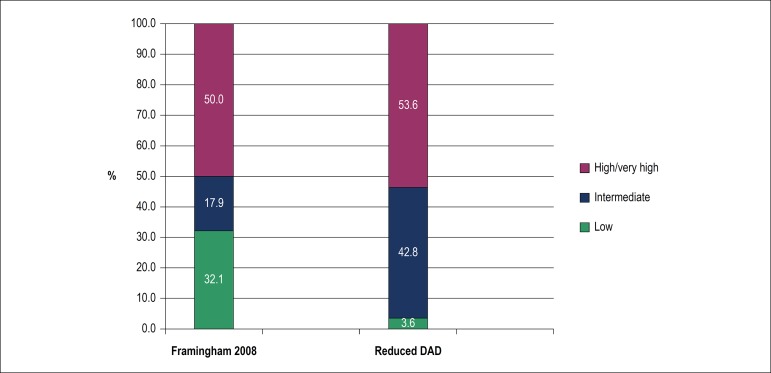
Classification of cardiovascular risk in 71 HIV-positive patients according to the Framingham 2008 and reduced DAD scores.

Among patients with subclinical atherosclerosis, 50% were classified as low or intermediate risk regardless of the score used. As for atherosclerosis stratified by Framingham 2008, 9/28 patients (32.1%) were classified as low risk and, by reduced DAD score, 12/28 (42.8%) were classified as intermediate risk (Figure 5).

**Figure 6:**
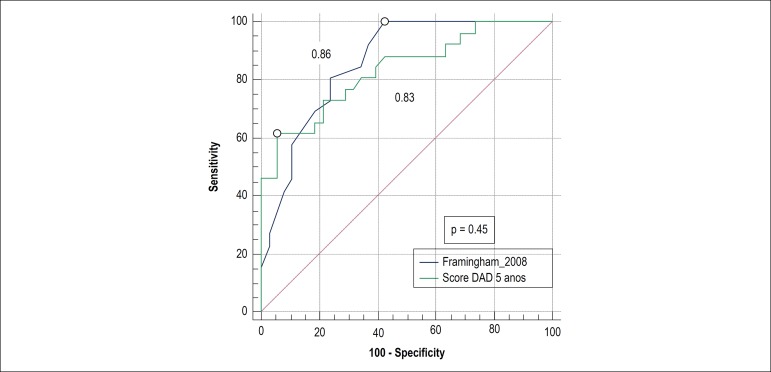
Comparison of ROC curves between Framingham 2008 and reduced DAD scores related to subclinical atherosclerosis (p = 0.46). Numbers represent the areas below the ROC curves. The circles represent the Youden Indexes of each score.

Analysis of discrimination of scores by comparison between ROC curves targeting subclinical atherosclerosis showed no significant difference between Framingham 2008 and reduced DAD scores (Figure 6), and the predictive accuracy is shown in Table 3.

**Table 3:** Predictive accuracy of Framingham 2008 and reduced DAD scores relating to the presence of subclinical atherosclerosis

Subclinical atherosclerosis	Sensitivity (%)	Specificity (%)	C-Statistics
Framingham 2008 > 3.9	100	57.8	0.86 (0.75 - 0.93)
DAD score > 3.3	65.5	92.8	0.85 (0.73 - 0.91)

DAD: Data Collection on Adverse Effects of Anti-HIV Drugs.

## Discussion

In this study, 71 HIV-positive patients under regular follow-up, diagnosed for more than one year, and under ART with immune reconstitution and viral suppression were evaluated. Most patients were classified as low risk by the Framingham 2008 score and intermediate risk by the reduced DAD score. There was a correlation between the medium-intimal thickening and the scores, high agreement between patients classified as high risk by both scores, although no significant difference was observed in the ROC curve score discrimination analysis. Although subclinical atherosclerosis was observed in 88% of patients classified as high risk by the reduced DAD score, subclinical atherosclerosis was found in 62.5% of patients classified as intermediate risk by the Framingham 2008 score.

Other authors have already compared risk stratification scores in HIV-positive patients,^8,14^ but to our knowledge, this is the first study to apply the reduced DAD score and assess the degree of agreement with Framingham’s score. Checking the accuracy and applicability of this tool is important because it has been developed for HIV-infected patients, and unlike DAD full, it does not use ART-related factors, which makes its use more feasible.

Regarding risk factors, it is important to highlight that, although diagnosis of dyslipidemia was reported in only 32.39% of patients, laboratory tests showed total cholesterol >200 mg/dL and/or triglycerides >150 mg/dL in 57.74% of the cases identifying significant difference between the diagnosis reported by the patient and the laboratory verification of changes in lipid profile. When considering dyslipidemia or changes in LDL-cholesterol, triglycerides or HDL-cholesterol, the frequency of dyslipidemia was 74.6%. These values reinforce the relevant presence of this risk factor in this population and the need for proper observation of criteria for diagnosis and treatment of these changes according to current guidelines.

The frequency of subclinical atherosclerosis reported here (39.4%) is similar to data from Falcão et al.,^15^ who found 42.6% of subclinical carotid atherosclerosis.^15^ Most patients evaluated were classified as low risk by the Framingham 2008 score and intermediate risk by the reduced DAD score, while the distribution of high risk was similar for both scores. Data from Nery et al.^14^ showed most patients classified as low risk by both Framingham and DAD full scores (94% x 74.2%), respectively, and both scores had a much smaller number of patients classified as high risk (2.8% and 2.1%), respectively, than in our sample.^14^

Although these scores are not used to estimate the presence of subclinical atherosclerosis, data from Jericó et al.^16^ show an increasing prevalence of subclinical carotid atherosclerosis according to cardiovascular risk category, with 26.6%, 35.3% and 76,5% for very low risk, low risk and moderate/high risk patients, respectively.^16^ Similar results were found in this study, where a positive correlation between medium-intimal thickening and score value was reported. These data are important and suggest that many patients classified as low and intermediate risk could be reclassified and managed as high risk due to the presence of subclinical atherosclerosis.

According to a recent publication, the Framingham’s score could underestimate cardiovascular risk in HIV-positive patients by showing a high prevalence of subclinical carotid atherosclerosis in patients sorted as low risk.^8^ The same authors suggest that the use of DAD full score allows a better association between risk stratification and the presence of subclinical atherosclerosis, and that other tools such as the verification of medium-intimal thickening may bring new information that can reclassify patients and reinforce the taking of measures of greater impact to control cardiovascular risk factors.

In our study, aggravating factors for reclassifying the Framingham 2008 score were disregarded as some guidelines recommend, which led to a higher number of individuals in the low-risk category. The inclusion of aggravating factors could overestimate the risk in 10 years and lead to a 10-fold increase in the proportion of patients classified as intermediate risk (3.2% to 39.9%).^14^

The use of the Framingham and DAD scores in a recent study conducted with 997 HIV-positive patients concluded that the Framingham score would attribute to this population a greater cardiovascular risk than the DAD full score, and that this could lead to overtreatment of patients and increased risk.^17^ Although the Framingham 2008 score underestimates the presence of subclinical atherosclerosis, in the reduced DAD score more than 50% of atherosclerosis patients are not classified as high-risk, suggesting that this score may also underestimate cardiovascular risk in HIV-positive patients. Another study evaluating 203 HIV-positive patients reported DAD score as having better performance than Framingham’s, and showed that its accuracy increases when CD4 lymphocyte parameters and albumin levels are incorporated. However, the detection of subclinical atherosclerosis was underestimated by both scores.^18^

To compare scores, a correlation was made between them and medium-intimal thickness, besides the calculation of degree of agreement between them, and ROC curve discrimination analysis. Scores were shown to be correlated with medium-intimal thickening. Kappa index was statistically significant and showed 49% of total agreement between scores, but substantially between high-risk patients. The intermediate risk category did not present statistically significant agreement and the low risk category presented low agreement. There was no statistically significant difference in the ROC curve discrimination analysis between scores. Importantly, these scores present differences in time and composition of type of predicted cardiovascular events. In addition, the Kappa coefficient may have limitations, and even low coefficients may show a good degree of agreement.^19^

In the present observational, cross-sectional study with a small and random sample, we performed risk stratification and the presence of subclinical atherosclerosis was assessed by carotid Doppler ultrasonography; however, the occurrence of clinical events was not evaluated. These data suggest that HIV-positive patients classified as intermediate risk by the Framingham 2008 and reduced DAD scores could be reclassified as high risk in up to 62.5% and 30.8% of cases, respectively, due to the presence of subclinical atherosclerosis detected by carotid Doppler ultrasonography. These results are relevant because high-risk patients demand more aggressive therapeutic goals and subclinical atherosclerosis could indicate the need for other classes of drugs such as platelet antiaggregants.

More appropriate risk stratification methods are highly desirable for this population, as this group, has risk factors inherent to chronic HIV infection itself in addition to increased risk of cardiovascular events, which leads to a systemic inflammatory process, and the use of ART increases the prevalence of metabolic syndrome. In addition to more accurate scores, new diagnostic tools or biomarkers may lead to stratification that allows better identification of high-risk patients. Thus, cardiovascular prevention measures can be reinforced not only by decrease in events, but also avoiding unnecessary use of medications that could cause adverse reactions and drug interactions.

## Conclusion

Although this study shows a correlation between the Framingham 2008 and the reduced DAD scores and medium-intimal thickening, as well as high agreement between patients classified as high risk, we could not find a statistically significant difference between them by ROC curve discrimination analysis. In addition, the results suggest that HIV-positive patients sorted as intermediate risk by Framingham 2008 and reduced DAD scores could be reclassified as high risk in up to 65.5% and 30.8% of cases, respectively, due to the presence of subclinical atherosclerosis detected by carotid Doppler ultrasonography.
